# A Narrative Review of Molecular, Immunohistochemical and In-Situ Techniques in Dermatopathology

**DOI:** 10.3389/bjbs.2024.13437

**Published:** 2024-12-17

**Authors:** J. A. Gabriel, N. Weerasinghe, P. Balachandran, R. Salih, G. E. Orchard

**Affiliations:** ^1^ St. John’s Dermatopathology Laboratory, Synnovis Analytics, St. Thomas’ Hospital, London, United Kingdom; ^2^ School of Health, Sports and Biosciences, University of East London, London, United Kingdom

**Keywords:** dermatopathology, PCR, NGS, molecular techniques, cutaneous disorders

## Abstract

Skin disorders pose a significant health burden globally, affecting millions of individuals across diverse demographics. Advancements in molecular techniques have revolutionised our understanding of the underlying mechanisms of skin disorders, offering insights into their pathogenesis, diagnosis, and potential targeted treatment. Furthermore, the integration of molecular diagnostics into clinical practice has enhanced the accuracy of skin disorder diagnoses. Polymerase chain reaction (PCR), next-generation sequencing (NGS), and other molecular assays have allowed for the detection of infectious agents, assessment of genetic mutations, and profile gene expression patterns with unequalled precision. These techniques have proven instrumental in distinguishing between subtypes of skin cancers, aiding treatment strategies and prognostic assessments. Moreover, molecular profiling is increasingly guiding the selection of therapeutic agents, ensuring a personalised and effective approach to managing skin disorders. The application of PCR has revolutionised the field by enabling the identification of microbial DNA (i.e., *Mycobacterium tuberculosis* and Epstein-Barr Virus) in skin infections and detecting specific genetic mutations associated with dermatological disorders (e.g., BRAF). DNA sequencing technologies, such as next-generation sequencing, have facilitated the elucidation of genetic variations and mutations in skin diseases (i.e., bullous disorders), paving the way for personalised treatment approaches. Gene expression profiling techniques, such as microarrays and RNA sequencing, have provided insights into dysregulated pathways and molecular signatures associated with conditions ranging from inflammatory skin disorders to cutaneous malignancies. Immunohistochemistry and fluorescence *in situ* hybridization have proven invaluable in determining protein expression patterns and detecting chromosomal abnormalities, respectively, aiding in the characterization of skin lesions in conjunction with the molecular data. Proteomic studies have contributed to understanding the intricate protein networks involved in dermatological conditions (i.e., psoriasis), while epigenetic analyses have shed light on the role of epigenetic modifications in gene regulation within skin cancer (i.e., Malignant Melanoma). Together, these molecular techniques have laid the groundwork for targeted therapies and precision medicine in dermatology, with implications for improved diagnostics and treatment outcomes. This review focuses on the routinely employed molecular techniques within dermatopathology, with a focus on cutaneous malignancies, autoimmune diseases, infectious diseases, and neonatal screening which can be implemented in the diagnosis and contribute to improved patient care.

## Introduction

Good clinico-pathological correlation is the basis for the study of skin diseases. There are few clinical investigations that are primarily based around the information acquired through the clinical appearance of a dermatological process often only employing naked eye assessments. However the information gained can be highly informative and directs differential diagnoses when correlated with morphological characteristics and patterns discerned through microscopic assessments. These subsequent microscopic assessments often utilise sophisticated techniques that inform on protein or RNA/DNA expression.

Molecular techniques progress this level of information and have steadily increased in terms of their applications within dermatopathology as they are often a prerequisite for any personalised medicine approach to patient treatment and management. Molecular techniques are based on the assessment of DNA, RNA and proteins to identify and classify disease states. But they also have a predictive and prognostic significance, ultimately playing a key role in the development of personalised medicine and patient management therapies. As the consistency and reliability of molecular assays are expanding in diagnostic settings, it is no longer a technology that is solely employed in large referral centres, but more appropriately utilised in most medical institutions. It is also the case that molecular techniques are now increasingly automated, increasingly reliable and accurate, coupled with a general affordability resulting in an increase in their application in point of care testing strategies [1].

In this review on molecular techniques in dermatopathology we discuss the key emerging technologies and discuss their applications within the context of cutaneous malignancies, autoimmune diseases, infectious diseases [2] as well as neonatal screening.

## Melanocytic Lesions (Malignant Melanoma)

Cutaneous melanoma is a malignant neoplasm that arises in the cells of the epidermis referred to as melanocytes. Melanocytes are responsible for producing melanin pigment and are predominately found in skin but are also found in the ears, eyes (Uvea), gastrointestinal tract, leptomeninges, genital, oral and sinonasal mucosal membranes [3]. The majority of melanoma cases are due to cutaneous malignancies (>90% diagnoses), with mucosal and uveal melanomas occurring less frequently (<15% diagnoses with country specific variation) [3]. The vast majority of cutaneous melanomas arise due to molecular changes induced by exposure to ultra-violet radiation, there are several rarer subtypes that are not [4]. The incidence of melanoma has increased in the recent decades with approximately 25 new cases per 100,000 in Europe and 30 cases per 100,000 in the USA [3]. The gold standard for the diagnosis of melanoma is through the morphological assessment of histological sections. However, with the development of molecular diagnostics techniques which assess and highlight genetic and epigenetic alterations, they have provided adjunctive diagnostic information for risk stratifying melanocytic lesions of uncertain malignant potential. Some good examples of this include fluorescent *in-situ* hybridisation (FISH), comparative genomic hybridisation (CGH), gene expression profiling, as well as targeted immunohistochemical assays.

### Comparative Genomic Hybridisation

CGH is utilised to detect chromosomal copy number variation (gains and loses) throughout the genome [5]. There are two main variations to this technique; classic and array-based CGH [5].

Classic CHG involves the analysis of lesional neoplastic tissue extracted from paraffin embedded histological sections [5, 6]. The tumour and normal human reference tissue samples are labelled using differently labelled fluorochromes, mixed in a 1:1 ratio [5–7]. The samples are then denatured and hybridised onto a substrate of normal metaphase chromosomes [5–7]. Following these steps the samples are visualised using fluorescent microscopy and analysed using computer software to compare the differential signal expression along the length of each chromosome [5, 6]. The relative wavelength observed is reflective of the proportion of tumour compared to normal DNA and can be used to detect gains and losses of DNA material [5, 8, 9].

Array-based CHG utilises arrayed artificial genomic clones as a substrate instead of normal metaphase chromosomes. This resulted in improved resolution, assay robustness and reproducibility compared to the classic CGH [5, 6]. On the array the dots correlate to genomic DNA from a specific locus and the number dots relates to the resolution [5, 6]. This assay depending on the platform can either be co-hybridizing the tumour and normal DNA onto the assay (like the classic CHG) or by hybridising only tumour DNA [5, 6]. With the latter method the copy number for a certain locus is determined by comparing the signal intensity of the tumour against a reference from a control series of non-tumour tissue [5, 6].

Single -nucleotide polymorphism (SNP) arrays is an alternative to CGH. SNP arrays utilises probed loci to bind to known SNPs and each genomic locus is represented by two spots on the array corresponding to the two alleles [5]. The SNP arrays provide an advantage due to its ability to detect loss of heterozygosity and detect allelic ratio [5]. As well as that SNP can also detect selected point mutations [5]. Recent protocol developments utilising molecular inversion probes (MIP) that require low quantities of tumour DNA from formalin fixed paraffin embedded (FFPE) tissue have been developed [5]. MIP are 40 bp in size, which allows for the evaluation of degraded DNA [5].

Although initial applications of CGH to melanoma primarily focused on metastatic tissue, subsequent literature has shown that CGH can also be beneficial in distinguishing benign melanocytic naevi and from primary cutaneous melanoma. Bastian et al were among the first to demonstrate this, showing that CGH had a sensitivity of 94.8% and a specificity of 90.4% in differentiating unequivocally benign melanocytic variants, including congenital, blue, and spitz naevi, from malignant melanoma [10]. They found that chromosomal aberrations were detected at a higher rate in melanoma compared to naevi [6].

### Fluorescence *In Situ* Hybridization (FISH)

Fluorescence *in situ* hybridization (FISH) is a technique used to detect alterations in chromosomal copy numbers at predetermined genomic loci [5]. This technique allows for the identification of various chromosomal changes, such as entire chromosome gains or losses, targeted loci alterations, loss of heterozygosity, and homozygous deletions [5]. The FISH methodology involves the generation and hybridisation of single stranded fluorescently labelled DNA probes which contain the gene of interest onto formalin fixed paraffin embedded, fresh or frozen tissue sections [5]. After the completion of several processing steps the results are examined and quantified microscopically [5]. FISH offers many advantages including less tissue requirement, faster turnaround times and direct visualisation of cells of interest [5]. However, the technique is limited to only targeted genes, potentially missing relevant alterations in genes not included in the assay [6].

A study by Gerami et al identified a panel of probes for 6p25 (RREB1), 6q23 (MYB), 11q13 (CCND1) and centromere 6 (CEP6) when used in combination assisted in differentiating between melanomas and naevi with a sensitivity of 86.7% and specificity of 95.4% [11]. In melanomas this probe set panel identified interchromsomal rearrangement in chromosome 6 with gains in 6p25 (RREB1) and loses in 6q23 (MYB), as well as common gains in 11q13 (CCND1) [11]. The FISH analysis is performed by evaluating 30 adjacent cell nuclei and calculating the percentage of nuclei where there is a gain in 6p25, 11q13 and a loss of 6q23 compared with CEP6 relative to validated cut-off values and the test result is considered positive if this is observed [11]. Although FISH exhibits high sensitivity and specificity in differentiating primary cutaneous melanomas from benign naevi [12, 13], its reliability in histologically ambiguous melanocytic tumours is variable [14].

### Gene Expression Profiling

Several innovative quantitative gene expression profiling platforms have emerged as supplementary diagnostic tools for evaluating melanocytic tumours. Examples of these technologies include the DecisionDx-Melanoma (Castle Biosciences, Friendswood, Texas), myPath Melanoma (Castle Biosciences, Friendswood, Texas) and Pigmented Lesion assay (DermTech, Inc., La Jolla, California) which utilises algorithmic analysis of RNA based gene expression profiles using tape stiped or biopsied patient tissue samples.

DecisionDx-Melanoma (Castle Biosciences, Friendswood, Texas) is a test produced by Castle Bioscience which is utilised for assessing the risk of metastatic disease in patients who have already been diagnosed with melanoma [15]. The test employs a messenger RNA based gene expression profile using reverse transcription polymer chain reaction [15]. The DecisionDx-Melanoma 31 GEP assay is made up of 28 genes that provides insight into prognostic potential and 3 control genes [15]. The gene panel was devised by identifying genes that over expressed and under expressed from publicly available dataset on metastatic melanoma [15]. The assay aims to provide insight into the likelihood of regional lymph node spread and overall disease survival [15]. By stratifying patients, it allows the identification of low risk patient groups that may not have to undergo invasive lymph node biopsies [15]. The assay classifies the patient results into two main groups, either as low risk (class 1) or high risk (class 2) [15].

The myPath Melanoma (Castle Biosciences, Friendswood, Texas) assay is a test that utilises a quantitative polymer chain reaction to aid in differentiating malignant melanoma from benign naevus in histologically ambiguous lesions [16]. The assay measures the expression of 23 genes (14 melanoma signature and 9 reference genes) and the level of signature genes and reference genes is evaluated [16]. The final result generated is a quantitative result ranging from −16.7 to 11.1 with a range between 16.7 and -2.1 classified as benign, −2.0 to −0.1 classified as intermediate and 0.0 to +11.1 classified as likely malignant [16]. Studies evaluating the myPath test have validated the GEP 23 as reliably differentiating ambiguous melanocytic lesions [16]. Clarke et al evaluated 1,400 melanocytic lesions as part of a prospectively submitted sample study [16]. The samples were evaluated histologically by three dermatopathologists and only samples which had diagnostic concordance of benign or malignant were included [16]. Results for sensitivity and specificity were generated to assess the score generated and the pathological diagnosis [16]. The gene expression signature differentiated benign naevi from malignant melanoma with a sensitivity of 91.5% and a specificity of 92.5% [16].

The Pigmented Lesion assay (DermTech, Inc., La Jolla, California) is a non-invasive gene expression test which utilises tape stripping of lesions to obtain stratum corneum from which RNA is then isolated [17]. From the isolated RNA the expression level of PRAME (preferentially expressed antigen in melanoma) and LINC 518 (long intergenic non-coding RNA 518) are evaluated [17]. Lesions expressing high levels of both PRAME and LINC518, either PRAME or LINC518 or neither correlates with high, moderate, or low risk of the lesions being malignant melanoma [17]. A study conducted by Gerami et al validating the PLA analysed 398 pigmented lesions (87 melanomas and 311 nonmelanomas), PLA was able to accurately differentiate with a sensitivity of 91% and a specificity score of 69% [18].

Molecular tests are used to predict response to immunotherapeutic drugs are usually utilised for patients with high stage tumour. Currently there are multiple therapeutic targets in melanoma with inhibitors which include the MAPK pathway, MEC and KIT [19–21].

The use of targeted therapies relies on the detection of an activation mutation on the *BRAF* gene [19, 20]. This is due to the fact that the use of these therapies without the genetic mutation can in turn lead to activation of the MAPK pathway [19, 20]. Another mutation encountered in melanoma is the NRAS- mutation which is found in 20% nonacral melanomas [22]. Currently, there are no effective targeted immunotherapy treatments for NRAS mutations, trials of MEK combined with cyclin dependent kinase 4/6 (CDK4/6) are be conducted to determine efficacy with this mutation [21]. A mutation encountered more commonly in acral and mucosal melanoma is the KIT mutation and these patients may benefit from the use of KIT inhibitors [21]. The detection of the *BRAF, NRAS and KIT* gene mutations are usually determined using next-generation sequencing and the positive detection is required for the consideration of inhibitory therapy [19–21].

### Immunohistochemistry

**FIGURE 1:**
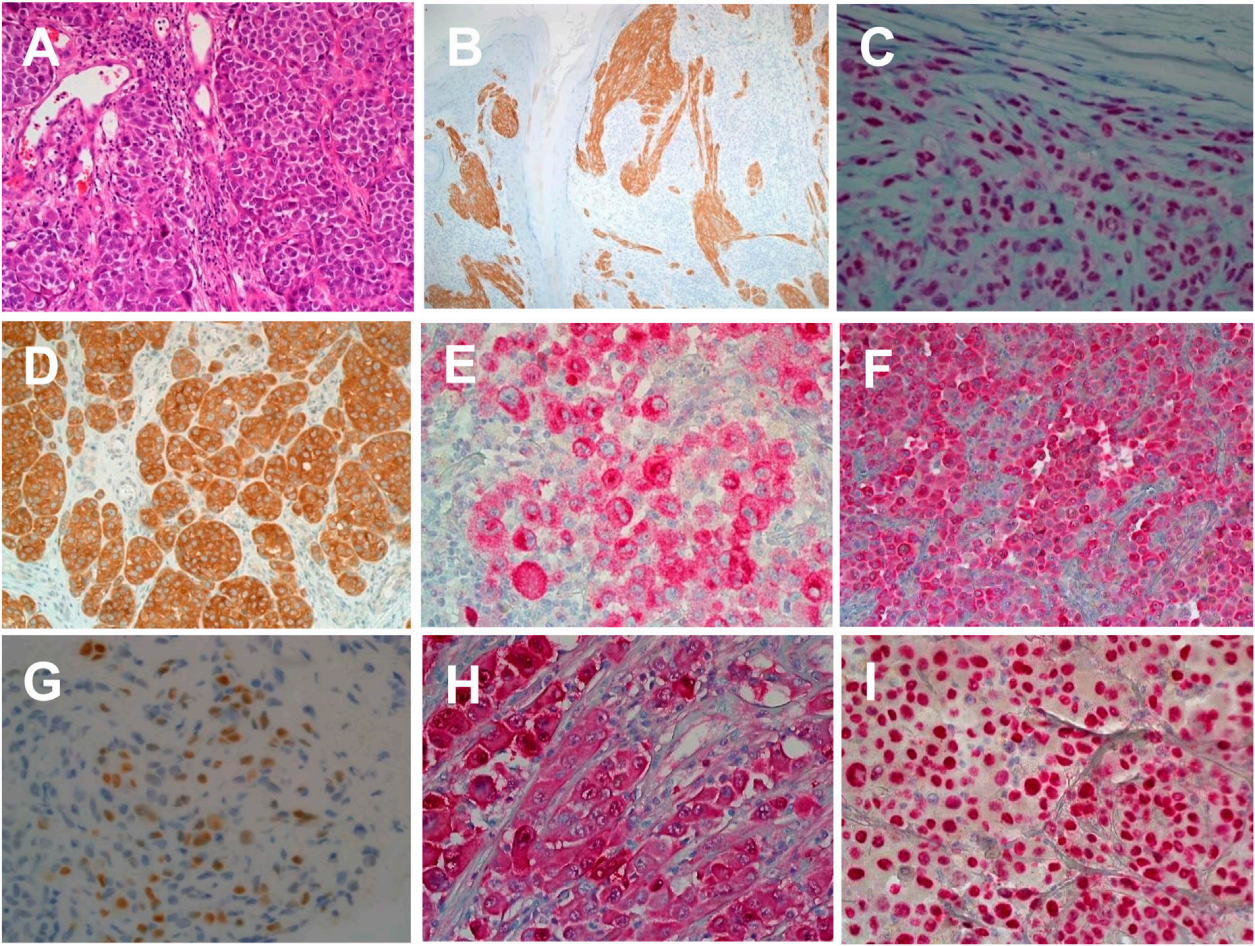
Immunohistochemical labelling of malignant melanoma in histological sections. **(A)** Haematoxylin and Eosin staining of skin section showing malignant melanoma (×20 magnification). **(B)** Anti-ALK1 expression in a melanocytic spitzoid tumour (×20 magnification). **(C)** Ant-BAP1 expression in in BAP1 inactivated melanocytic tumour (×20 magnification). **(D)** Anti-BRAFV600E expression in nodular malignant melanoma (×20 magnification). **(E)** Anti-HMB45 expression in superficial spreading melanoma (×20 magnification). **(F)** Anti-Melan A/MART1 expression in superficial spreading melanoma (×20 magnification). **(G)** Anti-PRAME expression in superficial spreading melanoma (×40 magnification). **(H)** Anti-S100 expression in superficial spreading melanoma (×20 magnification). **(I)** Anti-SOX10 expression in superficial spreading melanoma (×20 magnification).

The histological morphology of Melanomas can mimic a wide range of tumours including poorly differentiated carcinomas, lymphomas, sarcomas and germ cell tumours [23]. Melanoma cell morphologically can appear spindled or epithelioid with diverse cytoplasmic morphologies as rhabdoid, signet ring, clear cell, plasmacytoid and balling in appearance [23]. Immunohistochemistry (IHC) remains a very important adjunct tool in differentiating melanoma from other tumour types that they mimic. Commonly utilised markers for assessing melanocytic lesions include S100, PRAME, MART-1/Melan A, HMB45, Sox-10, MITF and Tyrosinase [23] (See Figure 1). Furthermore, IHC remains a useful tool in identifying genomic events that can aid in differentiating between tumour subtypes, these markers include BRAFV600E, Beta Catenin, PRKAR1, BAP1, ALK, PAN-TRK, NRASQ61R [24, 25]. The use of these markers is shown in Table 1.

**TABLE 1:** Immunohistochemistry tests used in identifying the subtype of melanocytic tumours [7].

IHC Marker	Genetic alteration detected	Diagnostic uses
ALK (Anaplastic Lymphoma Kinase)	Fusions	ALK rearranged Spitz tumours
BAP1 (BRCA1-Associated Protein 1)	Loss of function mutation and Loss of heterozygosity	Lost in BAP1 inactivated melanocytic tumours VS retained in Spitz tumours
β -catenin	*CTNNB1* activating mutation	Deep penetrating Naevus
BRAFV600E	*BRAFV600E* activating mutation	Negative in low risk spitz lesions VS positive in superficial spreading melanoma with spitzoid morphology
NRAS61R	*NRASQ61R* activating mutation	Negative in low risk spitz lesion VS positive in superficial spreading melanoma with spitzoid morphology
Pan-TRK	*NTRK1* and *NTRK3* fusions	NTRK-rearranged Spitz tumours
PRKR1A1	Loss of function mutation and Loss of heterozygosity	Pigmented epithelioid melanocytoma

## Cutaneous Lymphoma (Includes Discussions on Liquid Biopsy and NGS)

Lymphomas encompass a heterogeneous array of malignancies originating from the clonal proliferation of B-cell, T-cell, and natural killer (NK) cell populations within lymphocytes at different developmental stages. These malignancies collectively represent approximately 4.3% of all cancer cases in the UK; on average between 2016–2018 [26]. Lymphoma is classified into two main types; Hodgkin’s lymphoma (HL) and non-Hodgkin’s lymphoma (NHL). The estimated overall survival rate of 10 or more years for patients diagnosed with HL stands at 75% and NHL at 55% [26].

Up to 95% of Hodgkin’s lymphoma are classic Hodgkin’s lymphoma (CHL) and the rest are subdivided into four categories, each characterized by the presence of atypical cells known as Reed-Sternberg cells. These cells originate from B lymphocytes that undergo malignant transformation [27].

Although recent years have seen advancements in understanding the genetic makeup of CHL and nodular lymphocyte-predominant Hodgkin/B-cell lymphoma, mutational profiling currently lacks practical diagnostic significance [28]. Notably, modern protocols allow for the detection of B-cell clonality in a considerable subset of cases, rendering clonality studies ineffective in distinguishing Hodgkin lymphoma from other B-cell lymphomas with CHL-like morphology [28]. In contrast, identifying T-cell clonality and mutations characteristic of Follicular helper T-cell lymphoma may assist in distinguishing T-cell lymphomas with Reed-Sternberg cells from CHL [29].

Non-Hodgkin lymphoma (NHL) is a cancer of the lymphatic system. Diffuse large Bcell lymphoma and follicular lymphoma are among the most common subtypes in non- Hodgkin’s lymphoma, additionally there are more than 60 different types of nonHodgkin lymphoma [26].

T-cell and natural killer (NK)-cell neoplasms are relatively uncommon, comprising approximately 12% of non-Hodgkin lymphomas (NHL) collectively [30]. Despite their rarity, molecular assessment is commonly employed in clinical practice for most T-cell lymphoproliferations. This diagnostic necessity arises because T cells lack a definitive immunophenotypic marker of clonality, comparable to the kappa and lambda antigen receptor immunophenotyping in B cells, thereby necessitating the use of molecular techniques [31]. Specifically, clinical testing of T-cell lymphomas (TCL) typically involves two main categories of molecular changes: T-cell receptor (TCR) gene rearrangements and chromosomal alterations such as translocations, insertions, or deletions [32].

Additionally, as biopsies become smaller in size, distinguishing between neoplastic and reactive T-cell infiltrates based on immunomorphological criteria is becoming increasingly challenging [31]. To address this issue, molecular techniques such as multiplex polymerase chain reaction (PCR) assays to evaluate T-cell receptor (TCR) gene rearrangements have become widely adopted in daily clinical practice [31].

Multiplex PCR refers to a method employed to amplify numerous distinct genetic loci using multiple PCR primer pairs within a single reaction [28]. This technique enables the simultaneous addressing of various related inquiries about a specimen, eliminating the necessity for multiple individual PCR steps [28]. These individual PCR steps typically involve preparing separate reactions for each target sequence, each with its own set of primers and optimized conditions [28]. It is frequently utilized to confirm the presence of amplifiable nucleic acid in the sample [28].

**FIGURE 2:**
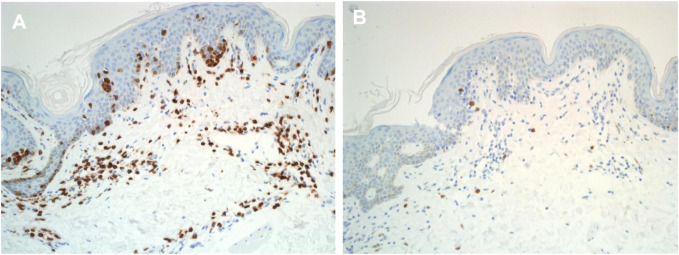
Immunohistochemical labelling of cutaneous lymphoma in histological sections. **(A)** Anti - Alpha Beta TCR expression in mycosis fungoides tissue section (X20) **(B)** Anti - Gama Delta TCR expression in mycosis fungoides tissue section (X20).

**FIGURE 3:**
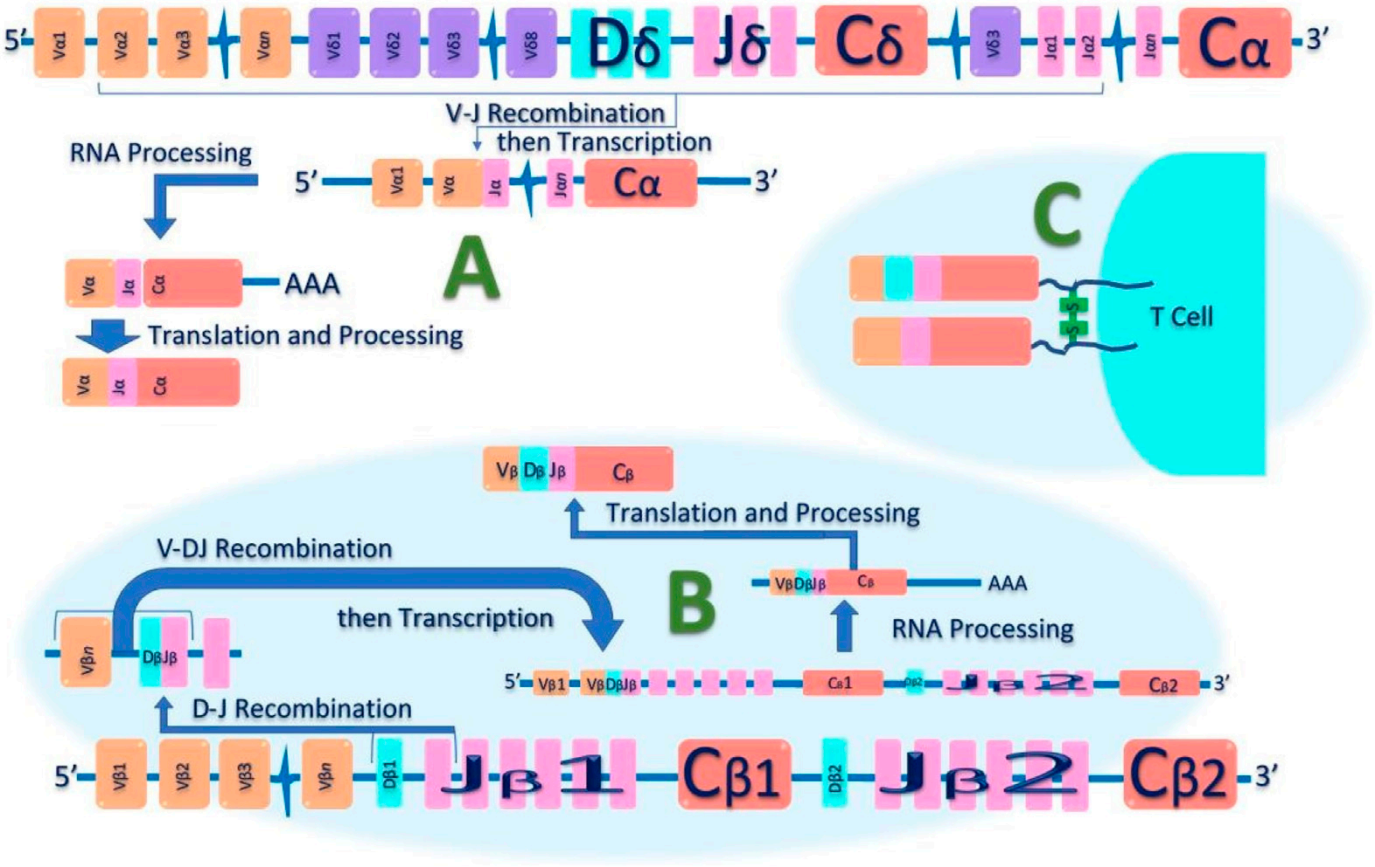
Schematic diagram of T-cell receptor (TCR) Alpha Beta Gene Rearrangement. Initially, in the undeveloped genome, the TCR’s genetic components are separated and unassembled. During T-cell development, these distinct genetic segments—variable (V), diversity (D), and joining (J)—are combined in various ways. Additionally, nucleotides at the junctions between these segments can be added or removed. This random assembly process, particularly in the complimentary determining region 3 (CDR3), creates such a vast array of possibilities that it's extremely unlikely for two T cells to have identical TCR nucleotide sequences. **(A)**: shows V-J recombination of the TCR-α chain DNA. **(B)**: Shows V-D-J recombination of the TCR-β chain DNA. **(C)**: shows heterodimer structure of αβ-TCR on the surface of T lymphocytes, that can serve as a unique molecular identifier for each T cell.

Most T lymphocytes possess α: β heterodimeric T-cell receptors (Figure 2); however, a subset expresses a unique γ: δ T-cell receptor comprised of distinct antigen-recognition chains, γ and δ, which are arranged in a γ: δ heterodimer [32]. These cells, characterized by such receptors, are referred to as γ: δ T cells. The TCR gene rearrangements occurs in these loci, and commonly analysed in dermatopathology using PCR [32] (Figure 3). Table 2 outlines the classification of Cutaneous TCell and B-Cell lymphomas, along with the known genetic abnormalities in each subset of cutaneous lymphoma. Additionally, clinical features and diagnostic immunohistochemical markers used in conjunction with molecular testing to confirm the diagnosis are provided. In most cases, immunohistochemistry (IHC) plays a crucial role in refining the diagnosis before molecular testing is conducted to pinpoint the specific diagnosis.

**TABLE 2:** WHO-EORTC Classification 2018 of Cutaneous lymphomas, involved genetic abnormalities and diagnostic immunohistochemical Markers [27–46].

WHO-EORTC Classification 2018	Gene/Translocation	Target/gene	Diagnostic immunohistochemical Markers
Cutaneous T-cell lymphomas			
Mycosis fungoides	CNV involve the 17p, 9p21 and 10q deletions and 17q amplification Somatic mutation in JAK/STAT	e.g. of some gene are: DNMT3A, ARID1A, CTCF, NCOR1, KDM6A, SMARCB1, ZEB1, PRKCB, PTPRN2, and RLTPR	CD3+, CD4+, CD8−, Cytotoxic proteins−, CD56−, αβ T cell lineage and EBV−
Mycosis fungoides variants • Folliculotropic MF • Pagetoid reticulosis • Granulomatous slack skin	Copy number variations (CNV) Somatic mutation in JAK/STAT		FMF: CD3+, CD4+, CD8-, with an elevated CD4:CD8 ratio (6–10:1); CD30 may be positive in large cell transformation PR: variable CD4, CD8, and CD30 expression; Ki-67/MIB1 may show active proliferation but is not specific GSS: CD4+/45RO+/30+; multinucleated giant cells, often CD68+, and may have surrounding CD1a+ cells
Sézary syndrome	CNVs Somatic mutation in JAK/STAT		CD3+, CD4+, CD5+/−, CD7−, CD25+/−, CD30+/−, CD56-, CCR4+
Adult T-cell leukemia/lymphoma	PLCG1,PRKCB,CARD11 and VAV1	CTLA4‐CD28 and ICOSCD28 fusions	CD3+, CD5+, CD45RO+, CD8+/−, CD25+/−, CD30+/−, CD7−, CD20−, CD79a-; elevated Ki-67 may be present in more aggressive forms of ATL
*Primary cutaneous CD30-positive lymphoproliferative disorders*			
• Primary cutaneous anaplastic large cell lymphoma	t(2;5)(p23;q35) 6p25.3 3q28 NPM1-TYK2 IL6-JAK-STAT mutation DNMT3A, TP53 mutation	ALK/NPM (ALK+ systemic) DUSP22/IRF4 TP63 JAK/STAT	CD3+/−, CD4−, CD8−, CD30+, Cytotoxic proteins+, CD56−, αβ T cell lineage and EBV−
• Lymphomatoid papulosis	IRF4/DUSP22 locus alteration		CD3+, CD4+, CD25+, CD30+, CD45RO+, CD56+/−, CD2−, CD3−, CD5−, CD7−. CD8 positivity, as opposed to CD4, is more frequently seen in Type D and Type E
Subcutaneous panniculitislike T-cell lymphoma			CD3+, CD4−, CD8+, Cytotoxic proteins+, CD56−, αβ T cell lineage and EBV−
Extranodal NK/T-cell lymphoma, nasal type	JAK-STAT mutation Gains in 8q24 [MYC]		CD3+, CD4-, CD8+ (surface CD3−), Cytotoxic proteins+, CD56+, NK or γδ T cell lineage and EBV+
Chronic active EBV infection	mutations in *DDX3X*		
*Primary cutaneous peripheral T-cell lymphoma, rare subtypes*			
• Primary cutaneous γ/δ T-cell lymphoma	STAT5B mutation SETD2 mutation	JAK/STAT	CD3+, CD4−, CD8−/+, Cytotoxic proteins+, CD56+, γδ T cell lineage and EBV−
• Primary cutaneous aggressive epidermotropic CD8-positive T-cell lymphoma (provisional)	CAPRIN1-JAK2 SELENOI-ABL1	JAK/STAT	CD3+, CD4−, CD8+, Cytotoxic proteins+, CD56−, αβ T cell lineage and EBV−
• Primary cutaneous CD4+ small/medium T-cell LPD (provisional)	Clonally rearranged TCR genes	Specific genetic abnormalities have not been described	CD3+, CD4+, CD8−, CD279/PD-1+, Cytotoxic proteins-, CD56−, αβ T cell lineage and EBV-.
• Primary cutaneous acral CD8+ T-cell lymphoma (provisional)	Clonally rearranged TCR genes	Specific genetic abnormalities have not been described	CD3+, CD4−, CD8+, Cytotoxic proteins- but TIA-1+, CD56−, αβ T cell lineage and EBV−
Primary cutaneous peripheral T-cell lymphoma, NOS	Clonally rearranged TCR genes	Specific genetic abnormalities have not been described	
Cutaneous B-cell lymphomas			
Primary cutaneous marginal zone lymphoma	T (14;18) (q32;q21) t (3;14) (p14.1;q32) 18q trisomy	IgH/MALT FOXP1/IGH FAS mutations	CD20+, CD79a+, BCL2+, BCL6−, CD5−, CD10−, CD16-
Primary cutaneous follicle center lymphoma	t(14;18)(q32;q21) 2p16.31 (amp REL 14q32.32 del 1p36 del	IgH/BCL2 (rare in cutaneous counterpart) TNFRS14 mutations	CD19+, CD20+, CD22+, CD79a+, PAX5+, BCL6+, CD10+/-, BCL2
Primary cutaneous diffuse large B-cell lymphoma, leg type	9p21 3p.14.1 6q del 8q24 3q27.3, 14q32 PDL1/PDL2-transl. 18q21.31–q21 ampl.	CDKN2A (or hypermethytation) FOXP1 BIMP1 MYC BCL6, IgH MYD88-mut, CD79B, CARD11, TNFAIP3/A20 (NF-κB) BCL2	CD19+, CD20+, CD22+, CD79a+, PAX-5+, BCL2+, IRF4/MUM-1+, FOXP1+
EBV-positive mucocutaneous ulcer (provisional)	Clonally rearranged IG and TCR genes		CD15+, CD30+, CD19+, CD22+, CD79a+, PAX-5+, EBV+, IRF4/MUM-1+, CD20+/−, CD10−, BCL6−
Intravascular large B-cell lymphoma	Mutations in MYD88, CD79B, SETD1B, and HLA-B and PD- L1/PD-L2 involving the 3' untranslated region		CD79a+, CD20+, IRF4/MUM-1+, CD5+/−, CD10+/−, CD29−, CD54−

Liquid biopsy has the advantage of overcoming tumour heterogeneity compared to contemporary testing methods. Traditional tissue biopsies may only capture a snapshot of the tumour’s genetic profile at a specific location, which may not fully represent the genetic diversity present throughout the tumour or across metastatic sites [47]. In contrast, liquid biopsy allows for the sampling of circulating tumour DNA shed from various tumour sites, providing a more comprehensive and dynamic view of the tumour’s genetic landscape [48]. This ability to capture genetic information from different tumour regions helps overcome the limitations of tumour heterogeneity seen with traditional tissue biopsies, ultimately leading to more accurate diagnosis and treatment selection [48].

Liquid biopsy samples are commonly analysed using Next-Generation Sequencing (NGS) technology; this allows for the comprehensive analysis of circulating tumour DNA (ctDNA) or other nucleic acids present in the liquid biopsy sample [49].

While ctDNA shedding can vary and may not capture every mutation, numerous studies validate its utility [47–55]. Research indicates that ctDNA often reflects a broad spectrum of genetic alterations present in different tumour regions and metastatic sites, offering a dynamic snapshot of tumour evolution [49]. Advances in Next-Generation Sequencing (NGS) and other technologies enhance the sensitivity and accuracy of ctDNA analysis, making it a reliable tool for providing a comprehensive and dynamic view of the tumour’s genetic makeup and informing on treatment decisions [49].

In the context of liquid biopsy analysis, NGS enables the detection and characterization of genetic alterations such as point mutations, insertions and deletions, copy number variations, and gene fusions in the circulating tumour DNA. This information can provide insights into the genetic makeup of the tumour, including its mutational profile, heterogeneity, and potential therapeutic targets [50, 51].

NGS-based liquid biopsy analysis offers several advantages, including high sensitivity and specificity, the ability to detect rare mutations, and the capacity for multiplexed analysis of multiple genes simultaneously [52]. Additionally, NGS allows for the monitoring of disease progression, treatment response, and the emergence of resistance mutations over time [52, 53].

Despite the advantages of liquid biopsies, there are several limitations. Liquid biopsies can experience issues such as inconsistent ctDNA shedding, leading to variability in detection and potential false negatives, particularly in early-stage cancers with low ctDNA concentrations [49]. Additionally, ctDNA assays may not capture all genetic mutations, potentially missing important alterations [48]. Overall, liquid biopsy has emerged as a powerful tool in precision oncology, enabling non-invasive monitoring of cancer dynamics and informing personalized treatment decisions [47–55]. These limitations highlight the need for ongoing research and technological improvements to enhance the reliability and availability of these diagnostic tools.

## Autoimmune and Genetic Skin Disorders

Conducting a thorough physical examination is a vital part of diagnosing blistering diseases, as it allows clinicians to evaluate the precise location, dispersion, and characteristics of the blisters themselves [56]. Additionally, dermatologists will examine a biopsy of the affected skin to analyse it under a microscope. Examining the skin sample enables detection of specific antibodies and other important indicators that provide evidence to support an accurate diagnosis [56, 57].

The diagnosis of autoimmune and genetic skin conditions has been advanced considerably in recent decades through the application of molecular biology techniques [56]. By enabling analysis at the DNA, RNA, and protein levels, modern molecular methods provide objective biological insight beyond visual symptoms. Techniques like PCR, DNA sequencing, microarrays, and mass spectrometry have illuminated causes and mechanisms in complex dermatological diseases [56].

In autoimmune disorders such as psoriasis, bullous pemphigoid, and lupus, molecular techniques assist in determining auto-antibodies involved in aberrant immune attacks on skin cells [56]. ELISA assays detect circulating autoantibodies in serum, while immunofluorescence microscopy localises autoantibody binding on skin biopsies. Identifying autoantibody profiles verifies diagnoses, reveals antigen targets, and guides treatment. PCR also measures inflammatory markers like cytokines to monitor disease severity [56, 57].

Beyond DNA, other developing molecular techniques further elucidate biological underpinnings [57]. RNA microarrays and sequencing reveal gene expression changes in diseased skin compared to healthy skin while mass spectrometry analyses the skin proteome (the entire complement of proteins) pinpoint dysregulated proteins [57]. Microbiome analysis examines microbial residents on skin that are related to autoimmune disorders [57].

These approaches provide precise, unbiased data about biological factors contributing to skin pathology [57]. Incorporating molecular biomarkers into diagnosis yields objective information that complements symptom-based observation. Molecular techniques also enable personalised medicine by matching treatments to an individual’s molecular profile [56, 57].

Epidermolysis bullosa (EB) refers to a group of rare genetic skin disorders that cause blistering and damaging of the skin in response to minor mechanical friction or trauma. There are four main types of inherited EB – EB simplex, junctional EB, dystrophic EB, and Kindler syndrome – with over 30 specific clinical subtypes [58]. EB displays substantial heterogeneity in symptoms, ranging from severe congenital blistering of the skin and mucous membranes that can impact lifespan, to very mild localised blistering such as nail dystrophy that begins later in life. In babies and adults, the blistering pattern and location may be distinctive enough to allow clinical diagnosis of the EB subtype. However, in newborns and milder cases, laboratory diagnostic testing is required to definitively determine the EB classification. Additionally, when EB occurs for the first time in a family, with no prior history, genetic testing is necessary to establish whether the inheritance pattern is autosomal dominant or recessive [58].

The four primary types of epidermolysis bullosa (EB) are categorised based on the ultrastructural level within the skin at which blistering, and separation occur. In EB simplex, splitting happens within the layers of the epidermis [59]. In junctional EB, it occurs in the lamina lucida layer. In dystrophic EB, cleavage takes place beneath the basement membrane zone in the uppermost dermis. Finally, in Kindler syndrome there is mixed-level blistering. An “onion skin” classification scheme for EB has been developed which sequentially considers the skin cleavage plane corresponding to EB type, clinical severity, inheritance pattern, and the specific molecular defect involved, including both the protein expression and disease-causing genetic mutations present [59].

In some subtypes of epidermolysis bullosa (EB), known as syndromic forms, the affected genes are expressed in tissues outside the skin, leading to involvement of other body systems and organs [60]. For example, muscular dystrophy can occur in EB simplex caused by plectin deficiency; pyloric atresia is seen in EB simplex with plectin deficiency and junctional EB with integrin α6β4 deficiency; cardiomyopathy is associated with KLHL24 or PLEC gene variants in EB simplex and with DSP and JUP variants in skin fragility disorders; lung fibrosis and nephrotic syndrome arise in junctional EB with integrin α3 subunit deficiency; connective tissue abnormalities occur with PLOD3 mutations; and nephrotic syndrome is seen with CD151 deficiency (Table 3). In these syndromic subtypes, the extracutaneous effects reflect expression of the defective genes in additional tissues, beyond simply the skin [59, 60].

**TABLE 3:** Classification and molecular characteristics of epidermolysis bullosa (EB) [52, 53].

Gene	Level of skin cleavage and ultrastructural anomalies	Relative protein expression	Types of pathogenic sequence variants	Protein	Inheritance
KRT5	Cleavage: basal keratinocyte cytoplasm; tonofilament clumping in EBS generalized severe; lack of tonofilaments in basal keratinocytes in AR EBS	Unchanged	Missense, nonsense, splice site, frameshift, in-frame (large) deletions or insertions	Keratin 5	AD
KRT14	Cleavage: basal keratinocyte cytoplasm; tonofilament clumping in EBS generalized severe; lack of tonofilaments in basal keratinocytes in AR EBS	Unchanged or absent	Missense, nonsense, splice site, frameshift, in-frame deletion or duplications	Keratin 14	AD, AR
PLEC	Cleavage: basal keratinocyte cytoplasm just above hemidesmosome s; diminutive hemidesmosome s	Plectin unchanged, absent or reduced with domain-specific antibodies	Missense, nonsense, frameshift, splice site	Plectin	AD, AR
KLHL24	Cleavage: basal keratinocyte cytoplasm; reduced tonofilaments in basal keratinocytes	Keratin 14 reduced or unchanged	Pathogenic variants in the translation initiation codon	Kelch-like protein 24	AD
DST	Cleavage: basal keratinocyte cytoplasm; diminutive hemidesmosome s lacking tonofilament attachment	BPAG1 (isoform e) absent	Nonsense, missense, frameshift, splice site	BPAG1	AR
EXPH5	Cleavage: basal keratinocyte cytoplasm; tonofilament aggregation in basal keratinocytes	Exophilin 5 absent	Nonsense, frameshift	Exophilin 5	AR
CD151	Cleavage: lower epidermis	CD151 absent	Frameshift, splice site	Tetraspanin 24	AR
TGM5	Cleavage: between stratum granulosum and corneum	Absent or reduced activity and expression of transglutamina se 5	Missense, nonsense, frameshift, splice site	Transglutam inase 5	AR
PKP1	Cleavage: suprabasal epidermal layers; hypoplastic desmosomes	Plakophilin 1 absent	Nonsense, frameshift, splice site	Plakophilin 1	AR
DSP	Cleavage: suprabasal epidermal layers; hypoplastic desmosomes	Desmoplakin reduced or absent	Nonsense, frameshift	Desmoplaki n	AR
JUP	Cleavage: suprabasal epidermal layers; hypoplastic desmosomes	Plakoglobin absent	Nonsense	Plakoglobin	AR

AD, autosomal dominant; AR, autosomal recessive.

For new-borns presenting with congenital skin absence, fragility, or blistering that could indicate epidermolysis bullosa (EB), prompt referral to a specialised EB diagnostic centre is recommended to establish a diagnosis [61]. The diagnostic workup should include acquiring a blood sample for DNA extraction, as well as a skin biopsy. Confirming the diagnosis can be achieved through immunofluorescence mapping (IFM) of skin samples using fluorescence-labelled antibodies, transmission electron microscopy (TEM) to examine skin ultrastructure, and/or direct genetic testing, depending on the centre’s capabilities. While genetic testing can provide a definitive result, IFM can yield a diagnosis within hours to guide urgent neonatal care [61]. Thus, IFM remains the preferred first-line approach currently, though genetic testing is increasingly accessible. In some complex cases, all three diagnostic modalities may be utilised to reach a conclusion. The goal is to leverage available resources to determine the EB subtype quickly and accurately [61].

For paediatric or adult patients who exhibit skin fragility and blistering consistent with epidermolysis bullosa (EB) subtypes, direct referral to a diagnostic centre for genetic testing can be appropriate once characteristic manifestations develop [61, 62]. The testing methodology chosen may involve next-generation sequencing (NGS) or Sanger sequencing (SS) depending on the circumstances. If both sequencing approaches fail to determine a genetic diagnosis, immunofluorescence mapping (IFM) and transmission electron microscopy (TEM) of skin samples may provide supplementary molecular and ultrastructural insights to elucidate the underlying basis for the skin fragility phenotype. In patients with clearer EB manifestations, proceeding straight to genetic analysis allows subtype classification, while IFM and TEM remain additional options when sequencing is inconclusive [61, 62].

Benign familial pemphigus (BFP) is an autosomal dominant skin disorder characterised by blistering and there are two main subtypes: BFP type I (Hailey-Hailey disease/HHD) and BFP type II (Gabriel’s disease) [63]. BFP type I is caused by mutations in the ATP2C1 gene which encodes a calcium ATPase and these mutations disrupt calcium homeostasis in keratinocytes leading to acantholysis (loss of cell adhesion). Approximately, 90% of HHD patients have ATP2C1 mutations while BFP type II is caused by mutations in M1S1 which encodes a desmosomal glycoprotein and these mutations impair keratinocyte adhesion through defective desmosome formation. The diagnosis of BFP’s include mutation screening of ATP2C1 and M1S1 genes by sequencing and immunofluorescence that shows loss of desmosomal proteins which is also visible on histology. Therefore, identifying causative mutations in adhesion proteins like ATP2C1 and M1S1 allows definitive diagnosis and classification of benign familial pemphigus subtypes [63].

Mucous membrane pemphigoid (MMP) refers to a group of chronic autoimmune blistering diseases primarily affecting the mucous membranes and skin. These diseases lead to progressive scarring and impairment [64, 65]. Laminin-332 is a key component of epithelial basement membranes, synthesised by keratinocytes. It plays an important role in dermal-epidermal adhesion and wound healing. Laminin-332 has a cross-like structure with three chains - α3, β3 and γ2 [64].

Patients with MMP have autoantibodies against the α3, β3 or γ2 subunits of laminin332. Immunoprecipitation of radiolabelled cultured keratinocytes has been the gold standard for detecting these autoantibodies [64, 66]. Alternatively, immunoblotting using keratinocyte extracellular matrix or purified laminin-332 can identify antibodies.

However, these techniques are time-consuming, labour-intensive, and restricted to specialised labs. Radio-immunoassays are highly sensitive and specific but cumbersome, expensive, and tightly regulated. ELISA systems using purified laminin332 or keratinocyte extracellular matrix have been developed as more accessible antibody detection methods [64, 67, 68].

However, significant barriers exist regarding cost, access, and practical integration into clinical dermatology. Much research has yet to translate out of specialised labs into widespread use. Additionally, the complexity of results requires collaboration between clinicians, researchers, and bioinformaticians to determine appropriate interpretation and application [69]. Therefore, histopathological analysis of these autoimmune diseases still remains to the gold standard technique.

## Infectious Diseases

Dermatopathology occupies a central position in the comprehensive diagnosis and management of skin conditions, extending to those with infectious aetiology. Whilst conventional culture-dependent methodologies and microscopic analyses have historically underpinned the diagnostic framework for most cutaneous infections, their effectiveness is circumscribed by inherent limitations. Specifically, microscopic examination through histochemical staining, exhibits diminished sensitivity and specificity, while culture-based strategies are characterised by prolonged turnaround times and are unsuitable for nonviable pathogens [70, 71]. The last few decades have seen the increased use of molecular techniques in the diagnosis of skin infections within the clinical and laboratory setting, through nucleic acid-based detection methods including PCR, *in-situ* hybridisation and sequencing of target pathogen DNA or RNA, detecting a wide range of infectious agents spanning, bacteria, viruses, fungi, and parasites in skin specimens [70, 71]. More importantly, these techniques have been particularly useful in the definitive diagnosis of often challenging and ambiguous infectious skin lesions, including tuberculosis, leishmaniasis, leprosy, lyme disease, and fungal infections [72–74]. Furthermore, the supplementary use of these molecular detection methods can also be applied to both fresh and formalin fixed paraffin embedded (FFPE) tissues, the latter of which relating to dermatopathology specimens [70, 72, 73, 75]. As a result, molecular techniques have become increasingly employed within the dermatopathology laboratory setting, having found use within the diagnosis of infectious or infection-related skin conditions.

In most cases, the conclusive diagnosis of cutaneous infections necessitates a comprehensive and interdisciplinary methodology. This combines clinical assessment with microbiological and microscopic investigations, alongside the established application of molecular assays aimed at identifying infectious pathogens from freshly obtained serological and tissue specimens [75, 76]. Specifically within dermatopathology, using routine histopathological techniques not only allows for the direct visualisation of infectious agents (histochemical stains) and infectious morphological hallmarks in tissues, but they also provide important context for differentiating between noninfectious and infectious aetiology, localisation of infectious agents within tissues, host response to infection, and also infectious disease progression [75]. In addition to H&E sections, a variety of ancillary tests in the form of special stains and IHC can be employed to directly demonstrate infectious agents within skin biopsies, aiding in diagnosis (Table 4). These ancillary tests offer a relatively inexpensive and quick method towards the microscopic demonstration of pathogens within tissues and are used routinely within dermatopathology. However, many of these ancillary tests often exhibit several limitations, namely a low sensitivity, arising due to low bacterial load within tissues, poor staining, as well as other technical considerations leading to required further testing [70, 71]. Whilst the examination of histopathological preparations forms the basis of dermatopathology, it alone, is not always sufficient towards the definitive diagnosis of skin infections [76]. It does however, at the very least, provide initial guidance and infectious work-up, correlating with other concurrent laboratory tests such as microbiological cultures and prompting for molecular testing [75].

**TABLE 4:** Key pathogens commonly found or relevant within dermatopathology 651 investigations with both conventional and molecular methods outlined [70–82].

Pathogen / Infection	Conventional diagnostic methods	Molecular diagnostic methods
Bacterial - Mycobacterium		
*Mycobacterium tuberculosis*	Histopathology & special staining by Ziehl-Neelsen (acid-fast). Tissue culture for M. *tuberculosis* with sensitivity testing.	PCR on fresh samples is recommended with further sequencing for antimicrobial resistance genes.
*Mycobacterium leprae*	Histopathology & special staining by Wade-Fite (modified acidfast).	PCR is a viable option on FFPE samples due to lack of culturing M. *Leprae*
Atypical *Mycobacteria*	Tissue Culture. Special staining by acid-fast stains are less useful during microscopy	PCR on fresh samples is recommended with sequencing for species differentiation
Bacterial - Spirochetes		
*Borrelia burgdorferi*	Serological studies	PCR on fresh (serologic) samples
*Treponema pallidum*	Serological studies. Histopathology with Anti- *Treponema* *pallidum* IHC is recommended and is more sensitive than special staining by Warthin-Starry.	PCR on fresh (serologic) samples. FFPE samples may also be used if fresh specimens cannot be obtained.
Bacterial - Other		
*Bartonella henselae*	Serological studies preferred. Tissue culture, although requires prolonged culturing time.	PCR on fresh (serologic) or. FFPE samples, with the latter to be used if suspected during histopathology work-up.
*Rickettsia rickettsii*	Serological studies.	PCR on fresh (eschar) skin sample. PCR on FFPE can also be useful
Viral		
Human Papilloma Virus, Human Herpesvirus-8, Herpes Simplex Virus 1 & 2, Merkel cell polyomavirus, Epstein–Barr virus.	Histopathology with adjunct IHC for viral proteins.	HPV, EBV – In-situ hybridisation (typically chromogenic) on FFPE sections would be suitable within the routine diagnostic setting. PCR may also be useful and can be used on FFPE tissues.
Fungal		
*Candida albicans, Histoplasmosis capsulatum,* Dermatophytes*,* *Aspergillus* species	Fungal culture & microscopy. In addition, IHC and special staining: periodic acid-Schiff (PAS) and Gömöri methenamine silver (GMS).	Pan-fungal PCR with subsequent sequencing, or species-specific PCR can be used on FFPE samples - ideally when fungal structures are observed histologically. Alternatively, in-situ hybridisation (alongside IHC) with species specific probes may also be useful in PAS/GMS positive sections.
Parasite		
*Leishmania spp.*	Histology with Giemsa special staining or in addition, anti- *Leishmania* IHC	PCR - broad-range or *Leishmania* genus PCR on Cultured samples which can then be followed by subspecies differentiation via restriction fragment length polymorphism (RFLP) or sequencing. PCR can also be carried out directly on fresh tissue samples and FFPE.

### Molecular Approaches for Infections in Dermatopathology

**FIGURE 4:**
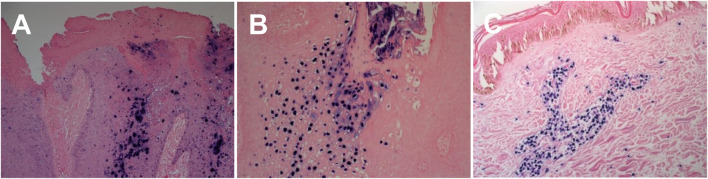
Chromogenic In - situ hybridisation labelling in histological sections. **(A)** HPV High risk expression in cutaneous genital wart lesion (X20) **(B)** HPV Low risk expression in cutaneous genital wart lesion (X20) **(C)** EBV expression in cutaneous EBV positive lymphoma tissue section (X20).

All pathogenic organisms have nucleic acid genomes which can be targeted by highly sensitive and specific molecular assays. Nucleic-acid-based amplification technologies (NAATs) such as PCR, along with several PCR-based variations, are some of the most widely used molecular methods for the detection of infectious pathogens and have seen much use over the last few decades [70–77]. The primary advantage of PCR-based methods is in the high level of sensitivity and specificity it provides, in a fast turnaround time, relative to other conventional methods relating to both microscopy and microbiological culturing. In addition to PCR, *in-situ* hybridisation (ISH) techniques can also be a viable alternative, particularly for viral infections [70, 77]. These ISH techniques utilise pathogen specific nucleic acid probes that can detect target pathogen DNA or RNA, in order to visualise infectious agents and their localisation within tissue sections, using chromogenic (CISH) or fluorescent labelling. Furthermore, CISH has the added advantage of being easily incorporated within the dermatopathology laboratory setting, utilising the same IHC platforms that are used routinely (Figure 4).

Sequencing technologies can also be employed on pathogen specific PCR amplicons, allowing for the simultaneous identification of pathogens at the species level in addition to the identification of drug resistant organisms [70, 75, 78, 79].

### Limitations of Molecular Techniques for Infections in Dermatopathology

Serology and fresh tissue cultures have traditionally served as the gold standard sample types for employing NAATs in pathogen detection. Whilst FFPE derived samples can be used for pathogen detection, it is associated with limitations that require further consideration for its appropriate use [70]. Molecular techniques such as PCR are not infallible and can be prone to false-positive findings when using FFPE derived samples, as a result of contamination during the numerous histological processing steps [70, 75]. Most importantly, FFPE derived samples are associated with false-negative findings that are a consequence of poor nucleic acid quality and lower yield. This is due to the degradation and alteration of nucleic acids due to resultant fixation and tissue processing leading to DNA fragmentation and formalin-induced sequence artefact [70, 75, 78, 79]. Furthermore, low presence of pathogens within FFPE tissues can also contribute to false-negative PCR results despite suggestive morphological features. As a result of these aforementioned limitations, a consensus review by Sunderkötter et al. outline the various indications, contraindications and key infectious scenarios to which NAATs should be appropriately requested as an adjunctive tool within the diagnostic workup, in order to prevent misinterpretation of results [70, 75]. In general, FFPE derived samples should be reserved for use as a “diagnostic rescue method” for NAATs, a scenario in which non-fixed samples can no longer be obtained from the patient, and or when slow growing or non-viable pathogens are suspected [70, 73, 74]. In these instances, adjunctive NAATs on FFPE sections can be vital to the diagnosis and worth conducting especially when infection is suspected at a later stage by the pathologist. Examples of infections that have clear indications for NAATs on FFPE tissues include; *Mycobacterium leprae*, cutaneous leishmaniasis, *bartonella spp*., rickettsiosis and *treponema pallidum* [70, 71, 73, 75, 80] Other key encountered infections within the dermatopathology setting such as cutaneous tuberculosis, atypical *mycobacterium*, *borrelia* and other fungal and viral skin infections, may benefit from NAATs when requested on FFPE tissues, albeit with greater reservation [70, 71, 73, 75, 81]. However, close histological and clinical correlation is vital when requesting NAATs on FFPE samples to minimise the impact of these aforementioned limitations and subsequent misinterpretation of results and misdiagnosis [70, 75, 81]. Table 4 provides a summary of relevant pathogens encountered in dermatopathology in which molecular techniques can be used to aid in diagnosis.

### Conclusion

There has been a steady rise in the application of molecular technologies within the field of dermatopathology. However there remains a requirement for extensive international collaboration to test applications and establish a broader base for clinical use generally [88–90]. The current literature is not robust enough in terms of large cohort studies to substantiated evidence for the application of most of these molecular biomarkers in a wider clinical setting [89, 90]. There is also a growing need to comply to *in vitro* diagnostic regulations (VDR) throughout Europe and this will increase the degree of rules and regulations on the use of these methodologies in diagnostic settings [82, 88].

The investment in novel equipment which can assist in the assessment of new molecular biomarkers is also constantly evolving with the development of innovative technology and near patient testing approaches [88]. Although these remain mainly applicable to the evaluation of infectious diseases currently [88]. The introduction of image analysis and the use of artificial intelligence, will accompany the rise in automation of complex molecular diagnostic assays. It will also improve the efficiency and enable faster assessment of patient material [91, 92].

The development of new and improved sampling techniques that are less invasive i.e., skin tapes, compared to conventionally employed tissue biopsies, are also set to expand [89]. It is also the case that these techniques will enable improved sensitivity and will rely on less patient DNA/ RNA being required for testing [89].

Of great interest will be the continual identification of new molecular biomarkers that are linked to prognostic outcomes, especially those associated with rare and unusual skin diseases such as epidermolysis bullosa (EB) [90]. These will expand our understanding of the disease processes still further.

Currently, many omics technologies have been utilised in the research setting to better understand cell populations in healthy and diseased states [88, 89]. Techniques such as spatial transcriptomics is a state-of-the-art technology with immense potential for future applications in various domains, such as medical research, cancer diagnostics, and therapeutic development [93, 94]. One of the most exciting uses is in cancer research, where it can analyse the intricate tumour microenvironment, revealing spatially distinct gene expression patterns that contribute to tumour growth and resistance to treatments [93, 94]. This insight can help identify new biomarkers for early detection and pave the way for highly targeted therapies, ultimately improving patient outcomes. This is particular key in many cancer and non-cancerous dermatological diseases [95, 96].

Moreover, spatial transcriptomics can enhance precision medicine by combining spatial gene expression data with other omics data, facilitating the creation of highly personalised treatment plans tailored to the unique spatial gene expression profiles of individual patients. This approach also has the potential to shed light on disease mechanisms, leading to more accurate therapeutic interventions. The potential integration of this techniques in routine practice could be a potential future development in dermatology.

Within the field of dermatology and more specifically dermatopathology there is a need to inform, educate and expand the repertoire of reliable and significantly validated tests that can be utilised within the diagnostic setting. There also needs to be clearer guidance and evidence-based research into potential biomarkers which will support the further development of new tests beyond the realm of research settings and into diagnostic prognostic and therapeutic applications. A number of new tests are yet to be validated in a diagnostic setting and as such remain academically interesting developments but as of yet not fully accepted in diagnostic practice. Ideally going forward there will be improved development in terms of new tests offering advanced sensitivity and specificity with the ultimate gain of improved patient care [97].

Molecular techniques represent a vital tool in the clinico-pathological correlation of skin disease states. The techniques and advances will enhance the information gleaned from conventional light microscope procedures that for so long have provided us with so much information on morphological criteria and disease processes and as a result will together continue to mould our diagnostic understanding (Table 5).

**TABLE 5:** An overview of the main immunohistochemical, *in-situ* hybridisation and molecular methodologies available, highlighting they key benefits and restrictions [83–87].

Name of technique	Benefits	Restrictions
Immunohistochemistry (IHC)	Detects at the light microscope level cellular compartment localisation of protein(s) expression Has application to both frozen tissue (i.e. immunofluorescence) and formalin fixed paraffin embedded tissue Cost effective Rapid turnaround times	Can lack high resolution imaging Limited options on multicolour chromogenic staining Prone to section detachment issues from slides, due to tissue or section preparation IHC slides will photo bleach over time Stringent controls needed to avoid false positives and false negative signal
	Reproducible and widely employed and can be fully automated Pivotal in certain tumour cancer pathologies for typing and classification of entities i.e. lymphoma typing.	interpretation. Due to a number of factors i.e. tissue processing, antigen retrieval or antibody concentration issues
In-situ hybridization (ISH)	Application on complex tissue types i.e. embryos. Higher resolution than IHC Useful to identify gene losses and duplications employing one chromogenic colour and gene splits and fusions (two colour chromogenic colours) Effective on formalin fixed paraffin embedded tissue and can be fully automated on IHC platforms Highly effective when employed in combination with IHC techniques i.e. Epstein Barr virus detection employing ISH in combination with lymphoma panel IHC or HPV detection employing ISH HPV high and low risk in conjunction with P16 expression in head and neck tumour pathology.	The procedures for in situ hybridization (ISH) are generally more complex and time-consuming compared to immunohistochemistry. ISH provides only semiquantitative results, allowing for the detection of relative changes in nucleic acid levels. Evaluating and identifying targets with low-copy number DNA or RNA can be challenging.
Polymerase Chain Reaction (PCR) DNA sequencing	High sensitivity and resolution technique can detect single genome copy changes i.e. base pair substitutions within fewer than 100 cells. Largely automated process Can be employed on material taken from formalin fixed paraffin embedded tissue blocks. Archival assessments employing PCR can be performed. Can be utilised as a technique to assess antimicrobial resistance	Relatively expensive compared to both IHC and ISH; thus, limited to small regions (target specific) Cannot distinguish between viable and nonviable cells: PCR can detect nucleic acids from both infectious and noninfectious pathogens, making it difficult to assess how harmful a sample is. PCR requires specific primers for each microorganism, which can be difficult for identifying mixtures of microorganisms. PCR can be inhibited by substances like metals found in environmental samples. Turnaround times are longer than IHC and ISH. Results need to be interpreted and assessed in the context of clinical symptoms.
PCR RNA sequencing	Gene Expression: RNA sequencing can identify changes in gene expression, including allelespecific variations, and can quantify significant differences in expression levels. Non-Coding Variants: RNA sequencing can reveal noncoding variants that may not	RNA sequencing generally has a longer turnaround time compared to most DNA sequencing methods. Choice of sampling methods can affect gene expression estimates and may also influence the sensitivity and specificity in
	be identified in traditional DNA sequencing methods. Fusion Genes: RNA sequencing is capable of detecting fusion genes without the limitations of predefined probe sequences. RNA Types: RNA sequencing allows for the characterization of various RNA types, such as mRNA and small regulatory RNAs. Reduced Noise: RNA sequencing generates data with less noise compared to microarray-based assays. New Transcripts: RNA sequencing can uncover new transcripts and coding regions that might not be detected through DNA sequencing.	detecting low-abundance transcripts.
Restriction fragment length polymorphism (RFLP)	Creates unique DNA profiles for individuals, making it especially effective for identifying common base pair variations. It is widely used and standardized across various applications. This method is reliable and relatively straightforward compared to other techniques.	More challenging to automate. Longer turnaround time compared to DNA sequencing. Results are gene-specific, requiring knowledge of common mutations associated with that gene. DNA degradation can lead to a higher likelihood of inconclusive results. Requires computer analysis and significant data storage capacity.
	It is also less susceptible to contamination from other DNA sources.	Read lengths are short, typically between 50 and 300 base pairs. Next-generation sequencing can identify various molecular abnormalities, though the clinical significance of many of these anomalies remains unclear.
